# Examination of Submandibular Muscle Activity During Swallowing of Different Materials by Multichannel Surface Electromyography in Healthy Males

**DOI:** 10.7759/cureus.73644

**Published:** 2024-11-13

**Authors:** Hirotaka Kato, Shinji Nozue, Yoshiaki Ihara, Atsumi Sunakawa, Kojiro Hirano, Akira Minoura, Akatsuki Kokaze, Kouzou Murakami, Yoshinori Ito, Toshio Watanabe

**Affiliations:** 1 Department of Oral Functional Rehabilitation Medicine, Showa University Graduate School of Dentistry, Tokyo, JPN; 2 Division of Oral Functional Rehabilitation Medicine, Department of Oral Health Management, School of Dentistry, Showa University, Tokyo, JPN; 3 Department of Otorhinolaryngology-Head and Neck Surgery, Showa University School of Medicine, Tokyo, JPN; 4 Department of Hygiene, Public Health and Preventive Medicine, Showa University School of Medicine, Tokyo, JPN; 5 Division of Radiation Oncology, Department of Radiology, Showa University School of Medicine, Tokyo, JPN; 6 Department of Respiratory Medicine and Allergology, Showa University School of Medicine, Tokyo, JPN

**Keywords:** healthy subjects, muscle activity, suprahyoid muscles, surface electromyography, swallowing

## Abstract

Introduction

Surface electromyography (sEMG), a widely used noninvasive technique for assessing muscle activity, measures muscle activity during swallowing. However, changes in the activity of each swallowing-related muscle, depending on the materials swallowed, remain unclear. Therefore, we investigated changes in muscle activity in the submandibular region using a seven-channel sEMG when swallowing different materials.

Materials and methods

This study included 38 healthy males (mean age = 38 years, standard deviation (SD) = 15.6 years). A seven-channel sEMG assessed and recorded submandibular muscle activity while participants swallowed saliva, 5 mL of water, and a capsule. The rising time (milliseconds (ms)), duration (ms), peak amplitude (millivolts (mV)), and integration (mV × ms) during swallowing were calculated from the sEMG recordings. Statistical analyses were performed using JMP Pro 16 (SAS Institute, Cary, NC). A one-way analysis of variance analyzed muscle activity for the different materials swallowed, and a t-test compared the muscle activities of each electrode. Bonferroni correction was applied, and p-values < 0.017 were considered statistically significant.

Results

No significant differences were observed in the rising time and duration of muscle activity between the different materials swallowed in any channel. However, significant differences in peak amplitude were found between the swallowed materials in certain channels: center-middle channel: saliva versus water, p = 0.0004; capsule versus water, p = 0.008; right-rear channel: saliva versus water, p = 0.0002; and left-rear channel: saliva versus water, p = 0.0005. Additionally, a significant difference in integration between the swallowed materials was observed in the center-middle channel (saliva versus water, p = 0.0035).

Conclusion

Our findings suggest that muscle activity in the submandibular center to the posterior region changes when different materials are swallowed.

## Introduction

Swallowing consists of three main stages: oral, pharyngeal, and esophageal. During the pharyngeal phase, the swallowing reflex occurs, transferring the food mass from the oropharynx to the esophagus. In this phase, the velopharyngeal function is activated, the fauces and larynx are closed, and the upper esophageal sphincter opens [1]. The laryngeal elevation, which facilitates both the closure of the larynx and the opening of the upper esophageal sphincter, is closely related to the elevation of the hyoid bone. The suprahyoid muscle group, involved in the elevation of the hyoid bone, consists of the mylohyoid muscle, anterior belly of the digastric muscle, stylohyoid muscle, posterior belly of the digastric muscle, and geniohyoid muscle. These muscles are intricately involved in swallowing [2].

Surface electromyography (sEMG) is widely used for noninvasive evaluation of muscle activity. sEMG has also been employed to analyze swallowing movements. Previous studies have explored the relationships between posture and dysphagia [3], muscle activity in the suprahyoid muscle group and dysphagia [4], and the effects of solution stimulation of the pharynx on the swallowing reflex [5]. Another study evaluated the relationship between dysphagia and muscle activity when swallowing foods with different physical properties [6]. These studies reported no differences in the muscle activity of the suprahyoid muscle group during swallowing [7].

However, despite the involvement of multiple muscles during swallowing, the specific locations of muscle activity within the suprahyoid muscle group have not been clarified. We hypothesized that the submandibular region coordinates muscle activity based on the characteristics of swallowed samples.

This study aimed to investigate the changes in suprahyoid muscle activity when swallowing boluses with different physical properties. We placed seven small electrodes in the submandibular region and examined muscle activity changes when swallowing different boluses, as well as the differences in muscle activity across various regions.

We believe that understanding the variations in muscle activity within the submandibular region will aid in electrode placement for devices that target the region, such as neuromuscular electrical stimulation (NMES). By clarifying these differences, we can select the primary target area in the submandibular region, enabling more efficient swallowing rehabilitation on swallowing muscles.

## Materials and methods

Participants

Thirty-eight healthy males (mean age = 38 years, standard deviation (SD) = 15.6 years) consented to participate in the study between November 9, 2021, and August 16, 2022. Exclusion criteria included a diagnosis of dysphagia, medication history affecting swallowing function, symptoms affecting eating and swallowing (such as sore throat, stomatitis, or toothache on the measurement day), dermatitis at the electrode placement site, individuals whose electrodes do not fit under the submandibular area, and participants who present atypical swallowing based on sEMG recording. The sample size was determined using the Z value from previous reports [8-10] and was set to exceed that value.

Electrode placement site

**Figure 1:**
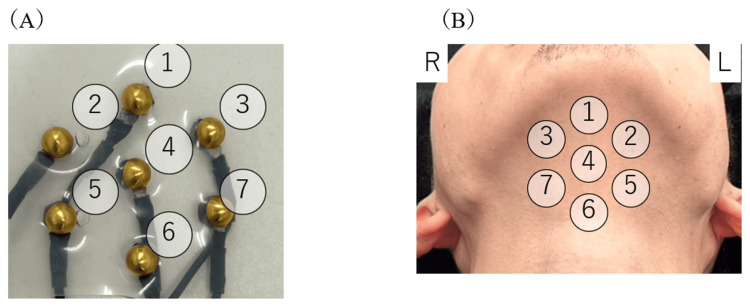
Location of electrodes and channel numbers (A) The electrode number as viewed from the skin contact surface. (B) The electrode numbers are shown with the electrodes affixed to the submandibular area.

Gold-plated steel electrodes, each with a diameter of 2 mm, were used to measure the sEMG [11]. The seven-channel electrodes were fabricated by inserting the connector part of each electrode into a transparent thin sheet with seven regular holes spaced 15 mm apart. The channels (CHs) were numbered, with CH1 being the centermost forward section and CH6 the centermost rear section. Figure 1A displays the CH number assignments. Participants were measured in an upright sitting posture and instructed to avoid movements that could affect the activity of the suprahyoid muscle group, such as speech and swallowing. After cleaning the submandibular skin with alcohol or benzalkonium chloride, the electrode was placed 1 cm posterior to the median of the medial aspect of the mandible (Figure 1B). The electrode application procedure, cleaning method, and ground electrode placement adhered to the sEMG for the Non-Invasive Assessment of Muscles project guidelines [12].

Swallowing tasks

The swallowing boluses consisted of saliva, 5 mL of water, and HF capsules (0.13 mL in volume) (Matsuya, Japan). This capsule was selected as the smallest of this product. Participants were instructed to swallow saliva first, followed by 5 mL of water administered into the oral floor via a syringe by the examiner, and finally, a capsule. No participants reported any residual sensation in the pharynx after swallowing 5 mL of water. The swallowing interval was long enough to consider muscle fatigue. If participants had difficulty swallowing the capsule, they were instructed to drink an additional 5 mL of water, and the data from those trials were excluded from the analysis.

Data analysis

**Figure 2:**
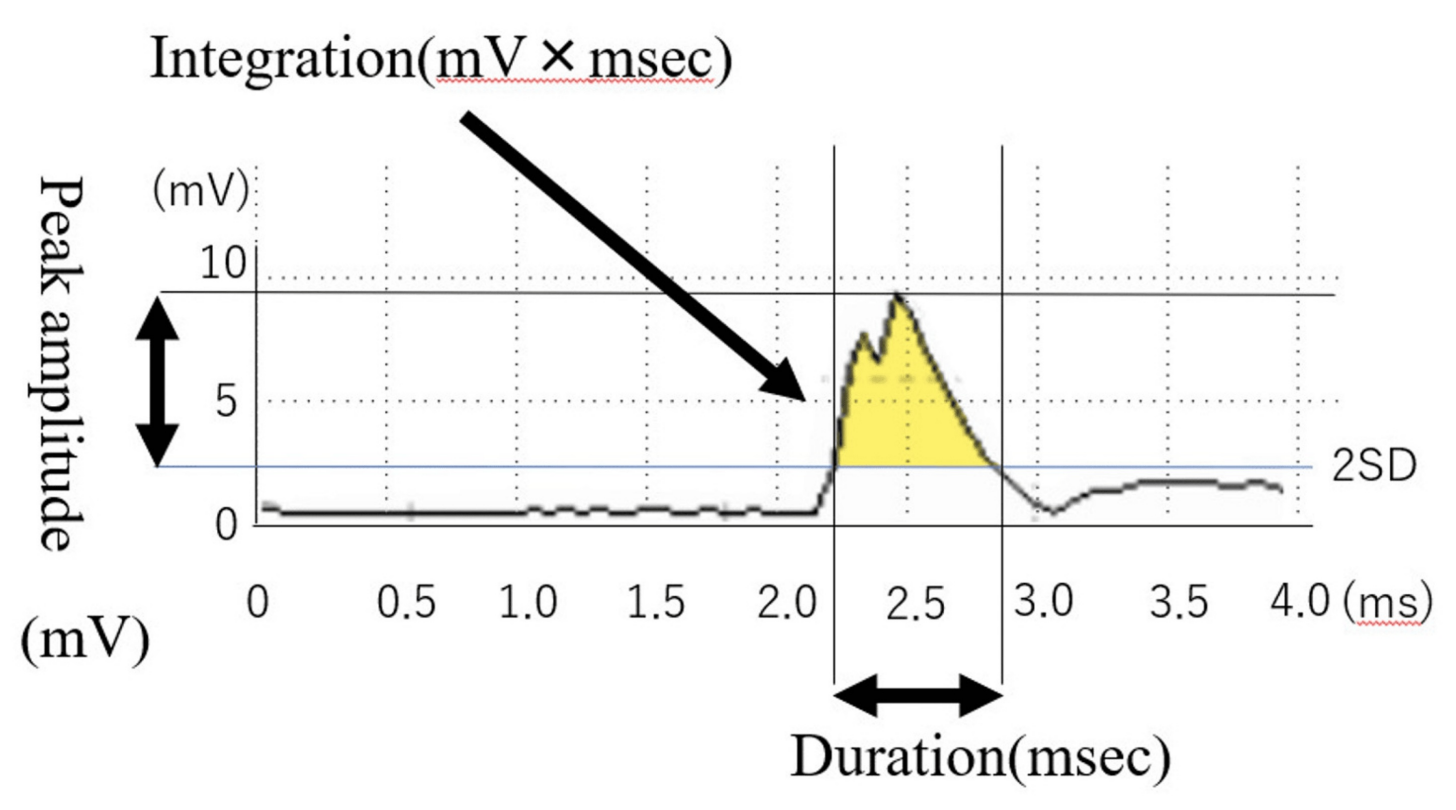
Rectified EMG waveform and duration, peak amplitude, and integration EMG: electromyography

Sample data signals were recorded on a personal computer using BIMUTAS-Video (64 bit) (KISSEI COMTEC Co., Ltd., Matsumoto, Japan) data acquisition software, with a sampling rate of 1,000 Hz. The sEMG data were analyzed for rising time (milliseconds (ms)), duration of muscle activity (duration (ms)), peak amplitude during swallowing activity (peak amplitude) (millivolts (mV)), and integrated muscle activity (integration) (millivolts × milliseconds) using BIMUTAS-Video. Rising time was determined from the raw waveform, defined as the time from the negative peak of the waveform to the positive peak amplitude. After analyzing the rising time, the captured raw waveforms were full-wave rectified (Figure 2).

The onset of muscle activity was identified as the point where the mean peak amplitude of the baseline exceeded +2 SD, and the offset was the last recorded point of muscle activity. The duration was defined as the time interval between the onset and offset. Peak amplitude was the maximum positive peak amplitude reached during the action potential. Integration was defined as the area under the swallowing activity waveform between the onset and offset, measuring the total discharge during swallowing.

Statistical analysis

Data analyses were performed using JMP Pro 16.0 (SAS Institute, Cary, NC). A one-way analysis of variance compared measurements obtained during saliva, 5 mL water, and a capsule swallowing. A corresponding t-test analyzed differences in muscle activity based on the swallowing boluses. A p-value < 0.05 was defined as statistically significant.

In addition, the p-values were corrected using the Bonferroni method to account for the multiplicity of the analysis (p < 0.017).

Ethics considerations

This study was approved by the Showa University Research Ethics Review Board (approval number: 21-088-A) on November 2, 2021. Before participating, participants were informed about the study's purpose and signed a paper consent form.

## Results

Rising time

Table 1 lists the rising times for each bolus obtained from all CHs. The mean rising time of CH1 was 0.082 ms (SD = 0.058) for saliva swallowing, 0.091 ms (SD = 0.049) for 5 mL water swallowing, and 0.108m s (SD = 0.870) for capsule swallowing. The mean rising time of CH2 was 0.115 ms (SD = 0.057) for saliva swallowing, 0.094 ms (SD = 0.047) for 5 mL water swallowing, and 0.110 ms (SD = 0.053) for capsule swallowing. The mean rising time of CH3 was 0.103 ms (SD = 0.046) for saliva swallowing, 0.097 ms (SD = 0.042) for 5 mL water swallowing, and 0.110 s (SD = 0.048) for capsule swallowing. The mean rising time of CH4 was 0.124 ms (SD = 0.043) for saliva swallowing, 0.103 ms (SD = 0.058) for 5 mL water swallowing, and 0.109 ms (SD = 0.055) for capsule swallowing. The mean rising time of CH5 was 0.117 ms (SD = 0.047) for saliva swallowing, 0.112 ms (SD = 0.046) for 5 mL water swallowing, and 0.106 ms (SD = 0.057) for capsule swallowing. The mean rising time of CH6 was 0.128 ms (SD = 0.055) for saliva swallowing, 0.108 ms (SD = 0.060) for 5 mL water swallowing, and 0.108 ms (SD = 0.061) for capsule swallowing. The mean rising time of CH7 was 0.116 ms (SD = 0.043) for saliva swallowing, 0.097 ms (SD = 0.062) for 5 mL water swallowing, and 0.108 ms (SD = 0.051) for capsule swallowing. No significant differences in the rising times were found among the different swallowed boluses across all CHs. 

**Table 1:** Rising time Rising time at each electrode obtained from each bolus p-values were calculated using the t-test. *p < 0.017

Channel	Rising time (ms)						p-value		
	Saliva		Water (5 mL)		Capsule		Saliva versus water	Saliva versus capsule	Water versus capsule
1	0.082	SD = 0.058	0.091	SD = 0.049	0.108	SD = 0.058	0.870	0.248	0.384
2	0.115	SD = 0.057	0.094	SD = 0.047	0.110	SD = 0.053	0.380	0.659	0.415
3	0.103	SD = 0.046	0.097	SD = 0.042	0.110	SD = 0.048	0.521	0.533	0.789
4	0.124	SD = 0.043	0.103	SD = 0.058	0.109	SD = 0.055	0.782	0.810	0.796
5	0.117	SD = 0.047	0.112	SD = 0.046	0.106	SD = 0.057	0.116	0.375	0.868
6	0.128	SD = 0.055	0.108	SD = 0.060	0.108	SD = 0.061	0.551	0.656	0.952
7	0.116	SD = 0.043	0.097	SD = 0.062	0.108	SD = 0.051	0.266	0.648	0.513

Duration

Table 2 lists the duration for each bolus obtained from all CHs. The mean duration of CH1 was 0.475 ms (SD = 0.079) for saliva swallowing, 0.476 ms (SD = 0.089) for 5 mL water swallowing, and 0.492 ms (SD = 0.078) for capsule swallowing. The mean duration of CH2 was 0.458 ms (SD = 0.096) for saliva swallowing, 0.509 ms (SD = 0.095) for 5 mL water swallowing, and 0.469 ms (SD = 0.085) for capsule swallowing. The mean duration of CH3 was 0.482 ms (SD = 0.080) for saliva swallowing, 0.478 ms (SD = 0.101) for 5 mL water swallowing, and 0.439 ms (SD = 0.557) for capsule swallowing. The mean duration of CH4 was 0.468 ms (SD = 0.092) for saliva swallowing, 0.485 ms (SD = 0.107) for 5 mL water swallowing, and 0.465 ms (SD = 0.087) for capsule swallowing. The mean duration of CH5 was 0.459 ms (SD = 0.077) for saliva swallowing, 0.439 ms (SD = 0.098) for 5 mL water swallowing, and 0.461 ms (SD = 0.070) for capsule swallowing. The mean duration of CH6 was 0.494 ms (SD = 0.097) for saliva swallowing, 0.467 ms (SD = 0.103) for 5 mL water swallowing, and 0.453 ms (SD = 0.100) for capsule swallowing. The mean duration of CH7 was 0.479 ms (SD = 0.082) for saliva swallowing, 0.449 ms (SD = 0.105) for 5 mL water swallowing, and 0.464 ms (SD = 0.079) for capsule swallowing. Similarly, no significant differences in duration were found among the swallowed boluses across the CHs.

**Table 2:** Duration Duration at each electrode obtained from each bolus p-values were calculated using the t-test. *p < 0.017

Channel	Duration (ms)						p-value		
	Saliva		Water (5 mL)		Capsule		Saliva versus water	Saliva versus capsule	Water versus capsule
1	0.475	SD = 0.079	0.467	SD = 0.089	0.492	SD = 0.078	0.586	0.935	0.310
2	0.458	SD = 0.096	0.509	SD = 0.095	0.469	SD = 0.085	0.600	0.935	0.547
3	0.482	SD = 0.080	0.478	SD = 0.101	0.439	SD = 0.089	0.557	0.256	0.113
4	0.468	SD = 0.092	0.485	SD = 0.107	0.465	SD = 0.087	0.969	0.209	0.182
5	0.459	SD = 0.077	0.439	SD = 0.098	0.461	SD = 0.070	0.253	0.148	0.779
6	0.494	SD = 0.097	0.467	SD = 0.103	0.453	SD = 0.100	0.776	0.295	0.233
7	0.479	SD = 0.082	0.449	SD = 0.105	0.464	SD = 0.079	0.051	0.147	0.886

Peak amplitude

Table 3 lists the peak amplitudes for each bolus obtained from all CHs. For central CH4, the median peak amplitude was 0.650 mV (SD = 0.822) for saliva, 0.430 mV (SD = 0. 333) for water, and 0.512 mV (SD = 0.617) for the capsule. The peak amplitudes for saliva and capsules were significantly higher than those for water (p = 0.0002 and p = 0.008, respectively). For CH5, the median peak amplitude was 0.864 mV (SD = 0.756) for saliva, 0.456 mV (SD = 0.386) for water, and 0.635 mV (SD = 0.888) for the capsule. The peak amplitude for saliva was significantly higher than for water (p = 0.0002). For CH7, the median peak amplitude was 0.509 mV (SD = 0.465) for saliva, 0.463 mV (SD = 0.282) for water, and 0.495 mV (SD = 0.502) for the capsule. The peak amplitude for saliva was significantly higher than that for water (p = 0.0005).

**Table 3:** Peak amplitude Peak amplitude at each electrode obtained from each bolus p-values were calculated using the t-test *p < 0.017

Channel	Peak amplitude (mV)						p-value		
	Saliva		Water (5 mL)		Capsule		Saliva versus water	Saliva versus capsule	Water versus capsule
1	0.584	SD = 0.985	0.398	SD = 0.766	0.595	SD = 0.905	0.506	0.333	0.232
2	0.482	SD = 0.501	0.384	SD = 0.512	0.513	SD = 0.441	0.126	0.977	0.191
3	0.448	SD = 0.438	0.359	SD = 0.346	0.438	SD = 0.789	0.226	0.283	0.069
4	0.650	SD = 0.822	0.430	SD = 0.333	0.512	SD = 0.617	0.0004*	0.071	0.0080*
5	0.864	SD = 0.756	0.456	SD = 0.386	0.635	SD = 0.888	0.0002*	0.398	0.021
6	0.579	SD = 0.898	0.527	SD = 0.822	0.511	SD = 0.457	0.101	0.069	0.069
7	0.509	SD = 0.465	0.463	SD = 0.282	0.495	SD = 0.502	0.0005*	0.327	0.017

Integration

Table 4 lists the integrations for each bolus obtained from all CHs. At CH4 in the center, the median EMG integral was 0.184 mV.ms (SD = 0.263) for saliva, 0.081 mV.ms (SD = 0.206) for water, and 0.112 mV.ms (SD = 0.256) for the capsule. The integration for saliva was significantly higher than that for water (p = 0.0035). A comparison between CHs revealed no significant differences in duration, peak amplitude, integration, rising time, or onset.

**Table 4:** Integration Integration at each electrode obtained from each bolus p-values were calculated using the t-test *p < 0.017

Channel	Integration (mV.ms)						p-value		
	Saliva		Water (5 mL)		Capsule		Saliva versus water	Saliva versus capsule	Water versus capsule
1	0.238	SD = 0.206	0.128	SD = 0.225	0.276	SD = 0.179	0.566	0.550	0.848
2	0.113	SD = 0.247	0.063	SD = 0.245	0.109	SD = 0.316	0.845	0.103	0.140
3	0.081	SD = 0.226	0.063	SD = 0.274	0.080	SD = 0.305	0.231	0.942	0.061
4	0.184	SD = 0.263	0.081	SD = 0.206	0.112	SD = 0.256	0.0035*	0.594	0.079
5	0.165	SD = 0.237	0.081	SD = 0.293	0.128	SD = 0.306	0.148	0.281	0.444
6	0.149	SD = 0.357	0.145	SD = 0.286	0.098	SD = 0.327	0.088	0.188	0.447
7	0.109	SD = 0.242	0.079	SD = 0.283	0.076	SD = 0.374	0.138	0.914	0.044

## Discussion

In this study, we hypothesized that the suprahyoid muscle group adjusts its activity when swallowing boluses with different physical properties and that muscle activity varies depending on the submandibular region. We compared duration and peak amplitude to examine muscle contraction force and integration to assess muscle activity. As a result, a significant difference was observed in the central and posterior submandibular regions. Therefore, it is possible to provide effective swallowing rehabilitation by placing electrodes in these areas using electrical stimulation therapy.

Procedure settings

This study included only male participants to reduce bias from differences of fat thickness in the submandibular region. It has been reported that there is a significant difference between men and women in fat thickness in this area [13]. It has also been reported that there is an effect on the distance between the electrodes and the submandibular muscles [14]. However, we did not measure the participant's BMI or fat thickness in the submandibular region. In addition, it has been reported that the muscle activity of laryngeal elevation during swallowing differs between men and women [15]. Additionally, we did not conduct detailed examinations of occlusion state, facial asymmetry, or cervical spine curvature, which can influence feeding and swallowing functions. However, since the boluses used in this study did not require mastication, the occlusion state likely had little effect. Moreover, the participants were healthy adults, so cervical spine curvature likely had minimal impact on the results. Regarding electrode positioning, the CH1 electrode was placed 1 cm behind the midline of the inner midline surface. Participants were excluded if electrodes on both sides did not fit into the submandibular region. Therefore, no differences between the left and right sides were expected. The swallowing boluses were inserted by the examiner, and the participants swallowed them according to instructions, minimizing the effects of the sample insertion method. Additionally, as each bolus was swallowed once by the healthy male participants, muscle fatigue effects were negligible.

A previous study indicated that the electrodes used in this study did not significantly differ from conventional electrodes in baseline, rising time and duration when swallowing saliva, 3 mL water, and 5 mL water. However, the peak amplitude of this study's electrodes was significantly higher than that of the conventional electrodes for all samples. No significant differences in the waveform characteristics of muscle activity were observed between this study's electrodes and the conventional ones [11]. Therefore, in this study, the electrodes were considered equivalent to conventional electrodes, except for peak amplitude.

Rising time

We hypothesized that the suprahyoid muscle group adjusts to boluses with different physical properties and that muscle activity varies by site; however, no significant differences in the rising time were found.

In a previous study, electrodes were attached to the bilateral submandibular and laryngeal regions of participants with Parkinson's disease, and submandibular muscle activity was analyzed by swallowing thin and viscous water as well as pudding three times each. No significant difference in the rising time was found among the boluses [16]. The result of this study had a similar tendency to previous reports. According to these results, rising time does not change with the physical properties of swallowed materials, regardless of the participant's swallowing condition.

Duration

We hypothesized that the suprahyoid muscles coordinate the swallowing of boluses with different physical properties and that muscle activity varies by site. However, no significant differences in duration were observed.

Previous studies have also reported that when comparing healthy adults using one set of electrodes for liquids of different properties and volumes, no significant differences in duration were found [3,17]. Similar results were obtained in this study. However, Koyama et al. measured muscle activity in both healthy adults and dysphagic patients swallowing boluses with different physical properties using two electrodes placed in the submandibular area. They reported that the submandibular muscle activity duration was significantly shorter in dysphagic patients than in healthy adults [18]. These studies have shown that the duration of swallowing jelly and thickened water is significantly shorter in dysphagic patients compared to healthy participants, and a significant difference in duration is observed with highly adhesive boluses. However, because highly adhesive boluses were not used in this study, no significant differences were observed in duration based on the physical properties of the boluses. Additionally, Koga et al. placed two electrodes on the right side of the neck in healthy adults and evaluated the submandibular and cervical muscle activity during pudding swallowing. They reported that the duration of the geniohyoid muscle activity was significantly longer in participants in their 70s and 80s than in those in their 20s [19]. Vaiman et al. divided healthy adults into three groups: young (18-40 years old), middle-aged (41-69 years old), and elderly (70 years old or older). They placed a set of electrodes in the submandibular region and instructed participants to swallow saliva, a sip of water, and 20 mL of water, and then evaluated muscle activity. In the elderly group, the duration was significantly longer with a sip of water and 20 mL of water compared to saliva [20]. A large degree of individual variation exists among older adults, and aging reduces the muscle fibers of the tongue, decreasing tongue pressure and dexterity [21]. The function of transporting a food bolus during swallowing is thought to decrease as tongue muscle strength diminishes [7]. When healthy adults were asked to swallow boluses with different physical properties in a swallowing contrast test, older adults demonstrated significantly longer durations for each sample compared to younger participants [22]. These findings suggest that older adults have a reduced ability to process differences in bolus physical properties and quantity, leading to variations in swallowing durations. One possible reason for the lack of significant difference in swallowing duration among the materials swallowed in this study may be that the age of participants was too young to exhibit such variations. Therefore, future research should focus on older adults.

Peak amplitude

Numerous studies have investigated the peak amplitudes of swallowing muscle activity with different bolus compositions and volumes.

Ko et al. studied the swallowing muscle activity of the submandibular region in participants younger than 60 years and those older than 60 years. They found that the peak amplitude for yogurt swallowing in participants under 60 was significantly higher than for those swallowing 5 mL of water [8]. Watts et al. measured the peak amplitude of cookies swallowed using two electrodes placed under the mandibular region. Healthy adults swallowed 15 mL of water, thickened water, pudding, and cookies, and the peak amplitude for the pudding swallowing was significantly higher than that of the water and thickened water [23]. Additionally, Inagaki et al. placed electrodes on the submandibular and lingual surfaces of healthy adults and reported that peak amplitude positively correlated with bolus viscosity [24].

These previous studies showed that the peak amplitude was higher for solids than liquids, larger boluses, and higher-viscosity boluses. In this study, saliva and capsules showed significantly higher values ​​than 5 mL of water in the CH at the central submandibular region.

In this study, the capsule was swallowed whole, consistent with the findings that the peak amplitude for solids is higher than that of liquids. The minimum amount of water used in previous studies was 10 mL, significantly higher than the amount of saliva used in this study. Therefore, the peak amplitude was considered higher when swallowing smaller amounts, such as saliva, compared to 5 mL of water. Li et al. placed an electrode under the jaw of healthy male participants and measured muscle activity while swallowing different amounts of water. They found no significant difference in the peak amplitude when different amounts of water were swallowed [9]. The results of this study and previous studies suggest that the amount of bolus swallowed significantly affects the peak amplitude of swallowing muscle activity.

Integration

In this study, the capsule showed significantly larger muscle activity than water in the right anterior, central, and most posterior submandibular regions. Inui et al. used two electrodes to measure changes in neck angle and sEMG integral values, reporting significant differences in the sEMG integral values ​​of the suprahyoid muscle group depending on posture [25]. The sEMG integral values ​​of the geniohyoid muscle were reported to increase when swallowing in the supine position compared to swallowing in the same position without a headrest, and sEMG integral values increased with added load [10]. In this study, all muscle activity recording was performed in the same upright sitting posture. Therefore, it is suggested that swallowing the capsule requires more effort than swallowing saliva or water, resulting in increased muscle activity. Additionally, this study found no differences between the left and right sides of the passage. However, Koga et al. reported significant differences between the left and right muscle activity during swallowing [7]. Previous studies have reported the existence of habitual chewing preferences, similar to the participant's handedness [26], suggesting that habitual bolus passing side during swallowing may exist, similar to chewing. Additionally, functional differences have been observed between habitual and non-habitual chewing sides [5]. However, this study did not investigate the left-right differences in motor function, masticatory muscle activity, and masticatory efficiency during chewing gummy jelly, as in previous studies. Future research should explore these aspects by simultaneously capturing frontal views during swallowing contrast examinations. Additionally, investigating the relationship between handedness and habitual chewing preferences is necessary.

Limitation

This study focused on the suprahyoid muscle group. However, since muscle courses were not confirmed by ultrasound before placing the electrodes, the recorded changes in muscle activity could not be attributed to specific muscles. Additionally, as the experiment involved only healthy male adults, different results may be obtained in patients with dysphagia, women, or older participants. Further studies should include healthy women and healthy elderly participants for broader statistical generalization. In addition, by including participants with dysphagia, it is necessary to compare their swallowing behavior with that of healthy adults and verify whether their swallowing behavior is different.

Furthermore, while this study compared the rising time, duration, peak amplitude, and integration of the boluses at each CH, comparisons between CHs are necessary to examine differences between the left and right sides.

## Conclusions

Using multichannel sEMG, submandibular muscle activity while swallowing three different boluses demonstrated significant differences in peak amplitude at the submandibular central area and significant differences in electromyogram integration ​​at the submandibular right anterior, central, and posterior regions. These results suggest that variations in muscle contraction in the submandibular central area, along with muscle activity in the submandibular right anterior, central, and posterior regions, are influenced by the properties of the bolus.
